# Centratherin Exhibits Antitumor Activity Against Glioblastoma Cells

**DOI:** 10.1007/s11064-025-04659-6

**Published:** 2026-02-07

**Authors:** Bruna Mafra de Faria, Fernanda Leme da Silva Pinheiro, Isabelle Medeiros, Jonathas F. R. Lobo, Andrew Magno Teixeira, Leandro Machado Rocha, Ricardo M. Borges, Maria Isabel Doria Rossi, Loraine Campanati de Andrade, Bruno Pontes, Luiz Gustavo Dubois, Luciana Ferreira Romão

**Affiliations:** 1 Instituto de Ciências Biomédicas, Universidade Federal do Rio de Janeiro, Rio de Janeiro, Brazil; 2 Instituto de Pesquisas de Produtos Naturais Walter Mors, Universidade Federal do Rio de Janeiro, Rio de Janeiro, Brazil

**Keywords:** Glioblastoma, Centratherin, Antitumor activity, Cell death

## Abstract

Glioblastoma (GB) is the most aggressive and lethal primary brain tumor, characterized by high proliferative, migratory, and invasive capacities, as well as marked resistance to apoptosis. Despite standard therapy with temozolomide (TMZ), prognosis remains poor, underscoring the need for novel therapeutic strategies. In this study, we investigated the antitumor potential of centratherin, a sesquiterpene lactone, in established GB cell lines and patient-derived GB cells (GBM02, GBM95). Centratherin significantly reduced cell viability in a dose-dependent manner, with IC50 values varying across GB cells, while exhibiting no cytotoxicity to healthy human astrocytes. Functional assays revealed that centratherin impairs cell proliferation, migration, and invasion, and alters cytoskeletal architecture, as evidenced by morphological changes, reduced actin and tubulin organization. Additionally, centratherin induced double-strand DNA breaks, increased γH2AX levels, and triggered cell death predominantly via necrosis, as demonstrated by LIVE/DEAD staining, Annexin V/PI flow cytometry, and ultrastructural analysis. Notably, this cytotoxic effect did not involve necroptosis, as RIP1 expression and Nec-1 sensitivity were unchanged. Furthermore, centratherin failed to sensitize GB cells to TMZ, suggesting distinct mechanisms of action, in spite of its remarked effect on inducing cell death in GB cancer stem-like cells. Overall, our findings highlight centratherin as a promising selective cytotoxic agent against GB, capable of inducing cell death and disrupting key malignant phenotypes, which may be advantageous for GB treatment.

**Supplementary Information:**

The online version contains supplementary material available at 10.1007/s11064-025-04659-6.

## Introduction

Glioblastoma (GB) is the most common and aggressive primary malignant tumor of the central nervous system, accounting for nearly half of all CNS malignancies [1]. Originating from glial cells, GB is characterized by rapid proliferation, diffuse infiltration into surrounding brain tissue, pronounced resistance to apoptosis, and marked intra- and intertumoral heterogeneity. Despite advances in multimodal treatment—including maximal surgical resection followed by radiotherapy and chemotherapy with temozolomide (TMZ)—prognosis remains dismal, with a median survival of approximately 15 months after diagnosis [2, 3]. These poor outcomes are largely due to GB’s complex molecular landscape, including frequent alterations in EGFR, PTEN, and other key regulators of cell cycle and apoptotic pathways [4, 5]. As such, there is a critical need for novel therapeutic strategies targeting the fundamental biology of GB.

Natural products have emerged as a promising source of new anticancer agents, and sesquiterpene lactones, a diverse class of secondary metabolites primarily derived from plants in the *Asteraceae* family, have shown particular potential. These compounds are known to modulate inflammatory and oncogenic signaling pathways, in part through the activity of their α-methylene-γ-lactone moiety, which reacts with nucleophilic thiol groups in proteins. This interaction can lead to the inhibition of key transcription factors such as NFκB, a central mediator of inflammation, survival, and tumor progression that is frequently hyperactivated in GB [6–8].

Centratherin is a sesquiterpene lactone first isolated in 1979 from *Centratherum punctatum*, a plant native to southeastern Brazil [9]. It shows a range of biological activities, including anti-inflammatory, antibacterial, antiparasitic, genotoxic, and antitumor effects [10, 11]. Preliminary findings indicate that centratherin reduces the viability of tumor cells, including GB cell lines U87 and U251 [11]. Despite these promising results, studies on centratherin antitumor effect remain scarce, and its effects on key tumorigenic processes such as cell proliferation, cytoskeletal organization, migration, DNA damage, and regulated cell death have yet to be fully elucidated.

In this study, we investigate the antitumor potential of centratherin in GB, focusing on its impact on cell proliferation, morphology, migration, and induction of DNA double-strand breaks leading to necrotic cell death. By expanding our understanding of centratherin’s mechanisms of action, we aim to assess its viability as a novel therapeutic agent for GB and contribute to the development of more effective treatments for this highly lethal disease.

## Materials and Methods

### Centratherin

Centratherin was extracted from the leaves of the plant *Eremanthus crotonoides* (DC.) Sch. Bip., belonging to the *Asteraceae* family, collected from the Jurubatiba Restinga National Park, Rio de Janeiro, Brazil as previously described [11]. The dried leaves of *E. crotonoides* were ground and macerated with 98% ethanol to obtain the ethanolic extract. This extract was then partitioned with hexane, generating the hexanic and hydroalcoholic phases. The hydroalcoholic phase was sequentially partitioned with CHCl, from which the CHCl_2_ extract was obtained. The CHCl_2_ extract was subsequently analyzed using UPLC-PDA-MS/MS chromatography, allowing its successful identification. To purify centratherin from the CHCl_2_ extract, fractionation was carried out using flash chromatography under reversed-phase silica gel [12]. The identification of centratherin was confirmed according to previously published ^1^H and ^13^C nuclear magnetic resonance (NMR) data in the literature. The 1H NMR spectrum of centratherin (shown in the Fig. S1) confirms its identity and indicates a high level of purity, estimated to be greater than 95%, based on the absence of extraneous resonances. However, a definitive quantification of purity (e.g., by qNMR or HPLC) was not determined at this stage.

### Cell Culture

Patient-derived GB cell lines GBM95 and GBM02, as well as cultures of healthy human astrocytes, were established at the Laboratory of Cellular Morphogenesis (Institute of Biomedical Sciences—UFRJ) from tumor specimens obtained at the Clementino Fraga Filho University Hospital, following the ethical guidelines of the Brazilian Ministry of Health, registered with the National Research Ethics Committee under record No. 2340, from 2001. GBM95 and GBM02 cell lines are *IDH* wild-type, negative for MGMT expression and express the EGFRvIII isoform [13, 14]. Healthy human astrocyte cultures were obtained through a previously established protocol [15], and the derivations of GBM95 and GBM02 from patient tumors were previously described [16]. The U87 cell line was obtained from the American Type Culture Collection (ATCC). All cells were cultured in Dulbecco’s Modified Eagle Medium supplemented with F12 (DMEM-F12—Cat No. 12400—Gibco), supplemented with glucose (35 mM), glutamine (2 mM), penicillin/streptomycin (0.5 mg/mL), fungizone (2.5 μg/mL), and sodium bicarbonate (3 mM) containing 10% Fetal Bovine Serum (FBS—Cat No. 12657—Gibco). The cells were maintained in an incubator at 5% CO_2_ and 37 °C until they reached confluence. Once confluent, the medium was removed, and 0.5% trypsin/ethylenediaminetetraacetic acid (EDTA) solution (Cat No. 15400—Invitrogen) or 0.02% EDTA (Isofar) in phosphate-buffered saline (PBS—NaCl, Na_2_HPO_4_, KH_2_PO_4_, and KCl—pH 7.4, 0.1 M) was added to detach the cells, which were then incubated for 5 min. Medium containing serum was added to inactivate trypsin, and the cells were centrifuged for 3 min at 300 g. After centrifugation, the supernatant was discarded, and the pellet was resuspended in medium with serum. The cells were counted in a Neubauer chamber and subsequently plated.

### Treatments

Centratherin was diluted in dimethyl sulfoxide (DMSO—Sigma) and then further diluted in medium at final concentrations of 0.5, 1.5, or 2.5 μg/mL (1.3, 4.0, or 6.6 µM). Taxol (Paclitaxel—Cat No. T7402—Sigma), used to induce cell cycle arrest in the M phase, was diluted in DMSO and then in medium at a final concentration of 1 µM. TMZ (Cat No. T2577—Sigma) was diluted in DMSO and then in medium at final concentrations of 100, 500, and 1000 µM. Necrostatin-1 (Nec-1—Cat No. N9037—Sigma) was diluted in DMSO and then in medium at a final concentration of 100 µM for GB cell treatment. The final DMSO concentration, determined by the 2.5 μg/mL centratherin treatment, was 0.001% v/v (~ 140 μM), a biologically insignificant level for cell assays. All control (CTRL) conditions were prepared using this same DMSO concentration, ensuring that any observed effects were attributable to centratherin rather than to the solvent.

### MTT Assay

GBM02 and GBM95 cells were plated at a density of 1.5 × 10^4^ cells per well, U87 at 3.0 × 10^4^ cells per well, and healthy human astrocytes at 7 × 10^3^ cells per well in a 96-well plate with medium containing serum and cultured in an incubator at 5% CO_2_ and 37 °C. Next, cells were treated with centratherin for 24 h. To assess viability, 3-(4,5-dimethylthiazol-2-yl)-2,5-diphenyltetrazolium bromide (MTT—Sigma—Cat No. M2128) was added to each well at a final concentration of 5 mg/mL and incubated for 2 h at 37 °C. After incubation, the supernatant was removed, 50 µL of DMSO was added to each well, and the plate was shaken for 15 min. Finally, absorbance was quantified using a plate reader (Victor 3 Perkin Elmer) at 570 nm. IC50 values were calculated as previously described [17]. Three independent experiments were performed in triplicate.

### Videomicroscopy

GBM02 cells were plated at a density of 4.0 × 10^5^ cells in a 35 mm diameter plate. Then, the cells were transferred to a CO₂ and temperature-controlled incubator (5% and 37 °C, respectively). The following day, the cells were treated with centratherin and placed in another incubator adapted to a Nikon Eclipse TE300 microscope (Nikon) with temperature and CO_2_ also controlled (37 °C and 5%, respectively). Over the next 20 h, phase-contrast images of the same field for each experimental condition were obtained every minute with the aid of a Hamamatsu C2400 CCD camera (Hamamatsu) attached to the microscope. The resulting image stacks were converted into videos, and analyses of proliferation, motility, and morphological changes were performed using ImageJ software. For proliferation analysis, the number of cells undergoing cytokinesis (the moment when one cell divides into two) in each video frame was quantified. Cell motility was performed as previously described [18]: at least ten cells per condition in each video were randomly selected, their positions tracked from the first to the last frame, and the trajectory traveled (in micrometers) was used to calculate cell speed. Morphological analyses were performed by counting cells with a round morphology in each video frame, as previously described [19]. To analyze the effect of centratherin on proliferation, migration, and morphology of GB cells, at least two independent videos were analyzed for each experimental condition.

### 3D Invasion Assay

GBM02 cells at a density of 2.0 × 10^6^ cells were plated in serum-containing medium in a non-adherent plate and immediately transferred to a shaker inside a CO₂ and temperature-controlled incubator (5% and 37 °C, respectively) to allow spheroid formation. The following day, these spheroids were plated as previously described [20]. For this, one spheroid was added to each well of a 96-well plate containing type I collagen matrix (serum-free medium, 1 mg/mL collagen I (BD Biosciences—Cat No. 354236), and 7.5 × 10⁻^3^ M NaOH). Centratherin treatment was added to each well, and at 0, 24, and 48 h, images of each well were acquired using the Nikon Eclipse TE300 microscope (Nikon). Using ImageJ software, the area occupied by the cells at 0, 24, and 48 h was quantified. Cell invasion analysis 24 and 48 h after treatment was performed by normalizing the occupied area to the time 0 measurement. Three independent experiments were conducted in triplicate. 

### Cell Cycle

GBM02 cells were plated at a density of 4.0 × 10^5^ cells per well in a 6-well plate with serum-containing medium, cultured in an incubator at 5% CO_2_ and 37 °C, and treated with centratherin the following day. At the end of the 24 h treatment, the supernatant from the wells was collected to harvest the cells that detached during treatment. The remaining cells were washed with PBS and then trypsinized. The trypsinized cells were then added to the tube containing the previously collected supernatant, centrifuged, and washed twice with PBS. Cell fixation was performed with 70% ethanol, as previously described [21]. The tube containing the cell pellet was placed on a vortex, and while being agitated, 1 mL of fixative was added drop wise. The fixed cells were then stored at − 20 °C. For flow cytometry analysis, cells were washed with PBS and incubated with 500 μL of FxCycle™ PI/RNase Staining Solution (Cat No. F10797—Invitrogen) for 30 min at room temperature in the dark. After incubation, cells were analyzed using the Attune NxT flow cytometer (ThermoFisher). The forward scatter (FSC) and side scatter (SSC) parameters were adjusted to locate the cells, and the population of interest was selected by excluding cellular debris. Then, cell doublets were eliminated from the analysis by selecting the population using FSC-area vs. FSC-height parameters. Finally, the PI fluorescence intensity, proportional to the amount of DNA in the cells, was analyzed using a histogram. The histogram displayed two peaks whereas the first peak corresponded to G1 phase cells; the second peak corresponded to G2 or M phase cells, and cells located between the two peaks were identified as being in the S phase of the cell cycle, as described in other studies [22]. Three independent experiments were conducted.

### Immunofluorescence

A total of 4.0 × 10^5^ GBM02 cells were plated in a 6-well plate with coverslips and cultured in an incubator at 5% CO₂ and 37 °C. The following day, the cells were treated with centratherin, and at the end of the treatment, cells were fixed with 4% paraformaldehyde (PFA—Isofar) for 15 min or with methanol for 5 min followed by 15 min with 4% PFA (for α-tubulin staining). After fixation, the cells were washed with PBS (pH 7.4, 0.1 M) and permeabilized with 0.2% Triton X-100 (Sigma) for 5 min. Next, nonspecific binding sites were blocked using 5% bovine serum albumin (BSA—Cat No. E588—AMRESCO) for 30 min. After blocking, the cells were incubated in a humid chamber with the primary antibody diluted in 1% BSA overnight at 4 °C or for 1 h at room temperature. After incubation, the cells were washed with PBS and incubated for 2 h in a humid chamber with the secondary antibody and/or Phalloidin, also diluted in 1% BSA. The cells were then washed and incubated with 4′,6′-diamino-2-phenylindole (DAPI) for 5 min to stain the nuclei. After another washing step, the slides were mounted with mounting medium (Fluoromount G). The stainings were visualized using a confocal microscope, and the images were analyzed using ImageJ software. For γH2AX analysis, fluorescence intensity was measured and normalized to the total number of cells in each image. For cytoskeleton analysis—including phalloidin, α-tubulin, and vimentin—filament organization was evaluated by quantifying anisotropy, as previously described [23, 24], using the FibrilTool plugin.

### Scanning Electron Microscopy

GBM02 cells were plated at a density of 4.0 × 10^5^ per well in 6-well plates with coverslips and cultured for 24 h in an incubator at 5% CO₂ and 37 °C. The following day, the cells were treated. At the end of the 24 h treatment, the cells were fixed for 1 h with a solution containing 2.5% glutaraldehyde diluted in 0.1 M sodium cacodylate buffer. The cells were then washed with 0.1 M sodium cacodylate buffer and post-fixed with 1% osmium tetroxide diluted in 0.1 M sodium cacodylate for 40 min in the dark. After incubation, the cells were washed again with 0.1 M sodium cacodylate buffer and dehydrated in a graded ethanol series (10–100%). Finally, the samples were transferred to a critical point drying apparatus (Bal-Tec), gold-coated using a metalizer (Bal-Tec), and then analyzed under a Quanta 250 scanning electron microscope (FEI Company), where micrographs were obtained. For scanning electron microscopy analysis, two independent experiments were performed.

### Transmission Electron Microscopy

1.7 × 10^6^ GBM02 cells were plated in 100 mm diameter plates. The cells were then treated. After the 24 h treatment, the cells were fixed for 2 h with a solution containing 4% PFA, 2.5% glutaraldehyde, and 0.1 M sodium cacodylate buffer, and washed with PBS pH 7.4, 0.1 M. The cells were then carefully scraped from the bottom of the plate and post-fixed with 1% osmium for 40 min in the dark. The pellet was washed with PBS and dehydrated in a series of increasing acetone concentrations (30–100%) and then infiltrated with a 1:1 acetone: EPON mixture overnight. Subsequently, the pellet was embedded in pure EPON, and the blocks were polymerized at 60 °C for 48 h. 70 nm sections were cut using an ultramicrotome (Leica EM UC7), contrasted with 2% uranyl acetate and 1% lead citrate, and visualized under a Spirit transmission electron microscope. One experiment was conducted for transmission electron microscopy analysis.

### LIVE/DEAD Assay

4.0 × 10^5^ GBM02 cells were plated per well in a 6-well plate. The following day, the cells were treated with centratherin, and at the end of the 24 h treatment, the cells were detached with trypsin, washed with serum-free medium, and then incubated with the dye from the LIVE/DEAD™ Fixable Green Dead Cell Stain Kit (Cat No. L23101—Invitrogen) for 30 min on ice in the dark. At the end of the incubation, the cells were washed with 1% BSA diluted in PBS and analyzed on the Attune NxT flow cytometer (ThermoFisher). The parameters of FSC vs. SSC were used to select the population of interest, excluding the cell debris, and the FSC-area vs FSC-height parameters were used to eliminate cell doublets. Histograms of the green dye were analyzed to identify live cells (negative for the dye)—the first peak of the histogram, and dead cells (positive for the dye)—the second peak. Three independent LIVE/DEAD experiments were performed.

### Annexin/PI Assay

4.0 × 10^5^ cells were plated in 6-well plate and cultured in an incubator with 5% CO2 at 37 °C. The following day, the cells were treated with centratherin, and at the end of the 24 h treatment, the supernatant from the wells was collected, the cells were trypsinized, and added to a tube with the collected supernatant. The cells were then centrifuged, washed with PBS buffer, resuspended in 96 μL of Annexin V buffer, and incubated with 1 μL of Annexin V and 12.5 μL of Propidium Iodide (PI) from the Annexin V FITC Early Apoptosis Detection Kit (Cat No. #6592—Cell Signaling) for 15 min on ice. After incubation, 300 μL of buffer was added, and the fluorescence was analyzed using the Canto II flow cytometer (BD Biosciences). The FSC vs. SSC parameters were used to select the population of interest, excluding cellular debris, and the FSC-area vs. FSC-height parameters were applied to eliminate cell doublets. Dot plot graphs of FITC and PI fluorescence were created to evaluate Annexin and PI staining for cell death analysis. Cells that are double-negative for Annexin V and PI are considered alive; cells positive only for Annexin V are in the early phase of apoptosis; cells positive only for PI are undergoing necrosis; finally, cells double-positive for Annexin V and PI may be considered in late apoptosis or necrosis [25]. Three independent Annexin/PI assays were conducted to assess the effects of centratherin on GBM cell death.

### Protein Extraction

GBM02 cells were plated at 4.0 × 10^5^ cells per well density in 6-well plates and cultured in an incubator with 5% CO_2_ at 37 °C. The following day, the cells were treated. After treatment, the supernatant from the wells was collected, and the cells were trypsinized. The trypsinized cells were added to a tube containing the collected supernatant, then centrifuged and washed with PBS. After washing, the cell pellet was resuspended in radioimmunoprecipitation assay (RIPA) buffer (50 mM Tris HCl pH 8.0, 150 mM NaCl, 1% NP40, 0.5% NaDeoxycholate, 0.1% SDS pH 7.5) in the presence of a protease inhibitor (Sigma—Cat No. P8340) or in tris-urea buffer (UTB) (75 mM TrisHCl, 9 M Urea, 1% Beta-mercaptoethanol). The sample was sonicated (2 pulses of 5 s with a 5 s interval) and then centrifuged at 8000×*g* for 10 min. After centrifugation, the supernatant was collected, sample buffer (Beta-mercaptoethanol, SDS, Tris 1 M pH 6.8, Glycerol, 0.1% bromophenol blue) was added, and the protein extract was boiled for 5 min at 95 °C.

### Western Blot

The protein samples were subjected to polyacrylamide gel electrophoresis (10% or 12%) at 110 V to separate proteins by molecular weight. The proteins were then transferred for 1.5 h at 0.1 A to polyvinylidene fluoride (PVDF) membrane (Bio-Rad), activated with methanol for 5 min. The membranes were incubated with 5% skim milk (Molico) for 1 h at room temperature under agitation to block non-specific binding sites and then incubated overnight at 4 °C with the primary antibody diluted in 1% milk. After incubation, the membranes were washed with tris-buffered saline (TBS) containing Tween-20 (TBST) (NaCl 150 mM, Tris 50 mM, Tween 20 0.1%, pH 7.5) and then incubated for 1 h and 20 min with the secondary antibody diluted in 1% milk. The membranes were washed with TBST. The bands were visualized by fluorescence emission using the Odyssey apparatus (LICor). The densitometry of the bands was performed using the UN-SCAN IT software. Three independent experiments were performed for the western blot analyses.

### Statistical Analysis

All values were expressed as mean ± standard error, except for the cell motility analysis performed through videomicroscopy (which was expressed in the form of a box plot). Statistical analysis of the results was performed using one-way ANOVA followed by Dunnett’s post-test using the GraphPad Prism software, comparing the experimental groups with the control. p Values < 0.05 were considered significant (*p < 0.05; **p < 0.01; ***p < 0.001).

## Results

### Centratherin Reduces GB Cell Viability Without Affecting Human Astrocytes

**Fig. 1:**
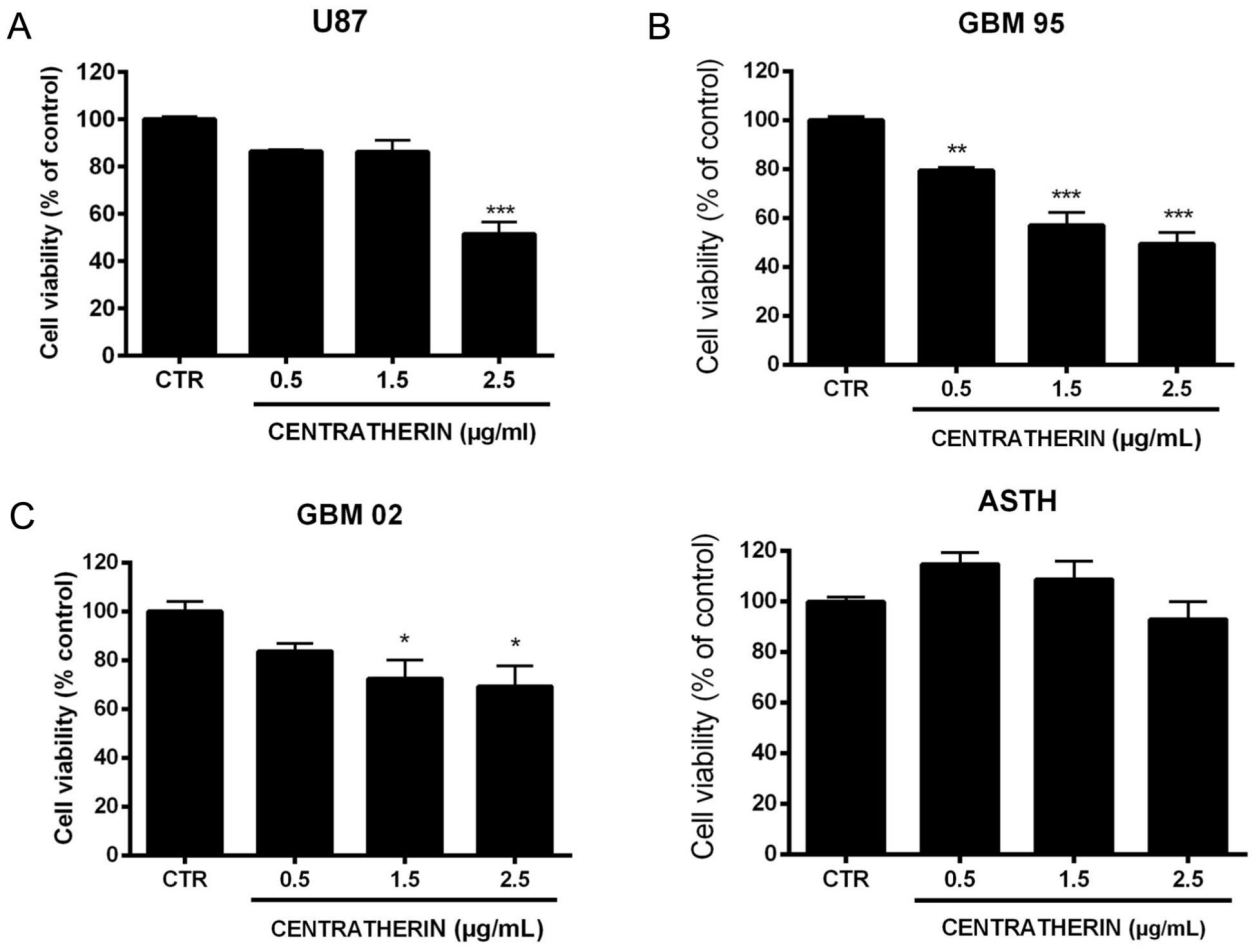
Centratherin decreases viability of GBM cells without affecting healthy human astrocytes. Cell viability of **A** U87, **B** GBM02, and **C** human astrocytes was evaluated by MTT assay after 24 h of treatment with 0.5, 1.5, or 2.5 µg/mL centratherin. Values represent the mean ± SEM of three independent experiments performed in triplicate. *p < 0.05, **p < 0.005 and ***p < 0.001 indicates comparisons between each concentration and the control

Previous studies have demonstrated that centratherin exerts antitumor activity against U87 and U251 GB [11]. To investigate its effects on a patient-derived GB cell lines (GBM95 and GBM02) established in our laboratory, we treated U87, GBM95 and GBM02 cells with 0.5, 1.5, and 2.5 μg/mL of centratherin for 24 h and assessed cell viability using the MTT assay. Centratherin significantly reduced GB cell viability, with decreases of 48.5% in U87, 50.6% in GBM95, and 30.6% in GBM02 cells at 2.5 μg/mL (Fig. 1A–C). To evaluate the selectivity of this effect, human astrocytes (ASTH) were treated with the same concentrations of centratherin. No significant reduction in viability was observed (Fig. 1D), indicating that centratherin selectively targets GB cells without inducing cytotoxicity in non-tumor astrocytes. Although GBM95 responded strongly to centratherin, GBM02 was selected for all further assays because this cell line has been extensively characterized in previous studies by our group, enabling more robust comparisons. In addition, GBM02 originates from a recurrent and clinically aggressive glioblastoma, making it a highly relevant model for mechanistic analysis.

### Centratherin Impairs GB Cell Proliferation, Motility, and Invasiveness, and Induces Morphological Alterations

**Fig. 2:**
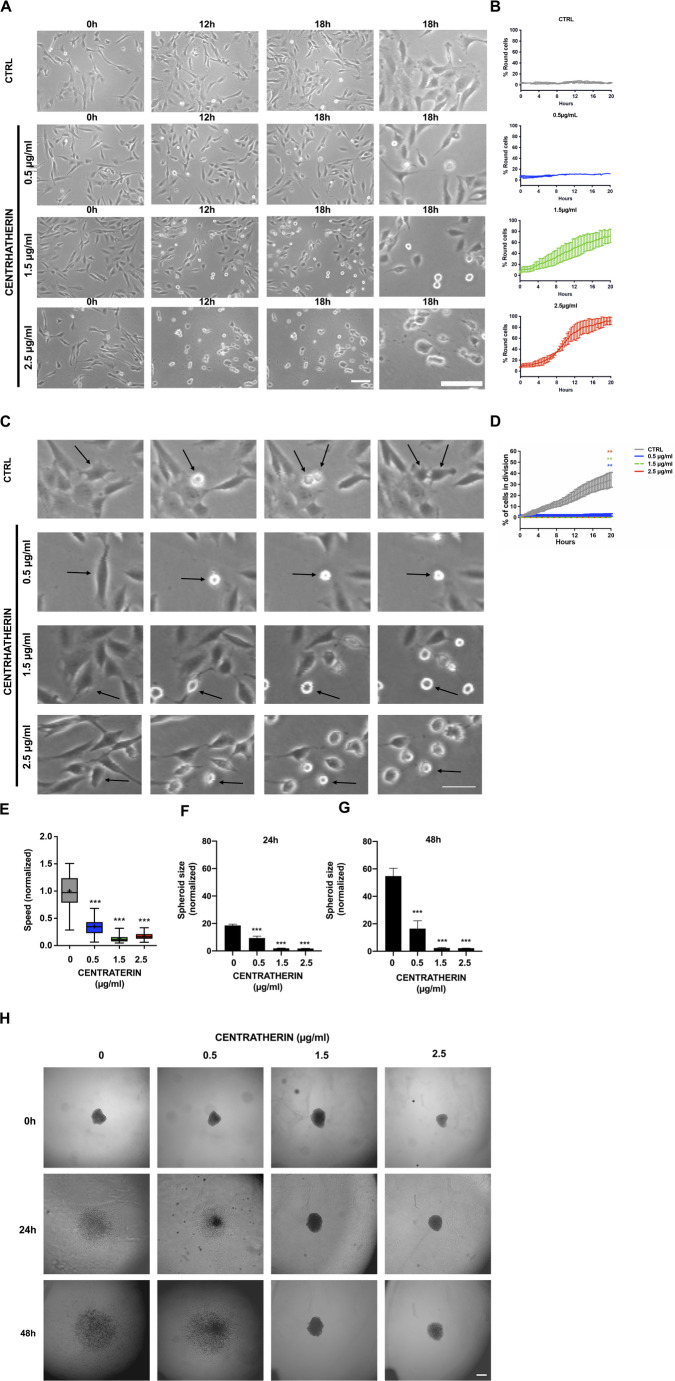
Centratherin impairs glioblastoma cell proliferation, motility, and invasiveness, and induces morphological alterations. Cells treated with vehicle (CTRL) or centratherin (0.5, 1.5, or 2.5 µg/mL) and analyzed by time-lapse video microscopy for 20 h. **A** Representative images show concentration- and time-dependent cell rounding and detachment; **B** quantification of rounded cells indicating 72.1 and 91.8% at 1.5 and 2.5 µg/mL, respectively. **C**, **D** Cell proliferation was assessed by quantifying division events during live imaging, revealing a marked reduction in the number of dividing cells, from 40% in the control group to 2.5% at 0.5 μg/mL, and complete inhibition of division at 1.5 and 2.5 μg/mL. Arrows indicate round cells. **E** Cell motility analysis showed a significant decrease in migration speed in a dose-dependent manner, with reductions of 65%, 87%, and 83% at 0.5, 1.5, and 2.5 μg/mL, respectively, compared to controls. Invasive capacity was evaluated using a 3D spheroid invasion assay in collagen I matrix. **F**, **G** Representative images at 24 and 48 h and **H** corresponding quantifications show that centratherin substantially impairs invasion. At 24 h, the invaded area was reduced by half at 0.5 μg/mL, and by approximately tenfold at 1.5 and 2.5 μg/mL; at 48 h, the reduction reached threefold at 0.5 μg/mL and around 24-fold at higher concentrations. Scale bars: **A** 100 µm, **C** 50 µm, and **H** 25 µm. All data represent mean ± SEM (n = 3). Statistical significance: *p < 0.05, **p < 0.01, ***p < 0.001 compared to control

We further investigated the antitumor properties of centratherin using time-lapse video microscopy to assess its impact on GB cell proliferation, motility, and morphology (Videos S1–S4). Centratherin induced marked morphological changes, causing GB cells to detach from the culture surface and adopt a rounded shape in both a concentration- and time-dependent manner. Treatment with 1.5 and 2.5 μg/mL resulted in 72.1 and 91.8% of cells displaying a rounded morphology, respectively (Fig. 2A, B and Videos S3, S4, compared to Video S1, control). Proliferation analysis revealed that centratherin significantly suppressed GB cell division: the percentage of dividing cells dropped from 40% in control to 2.5% at 0.5 μg/mL and 0% at 1.5 and 2.5 μg/mL (Fig. 2C, D and Videos S2–S4 compared to Video S1, control). Cell tracking analyses revealed that centratherin also reduced cell motility in a dose-dependent manner. Even at 0.5 μg/mL, cell migration speed was reduced by 65%, with further reductions of 87 and 83% at 1.5 and 2.5 μg/mL, respectively (Fig. 2E and Videos S2–S4, compared to Video S1, control). To evaluate invasive potential, we conducted a 3D spheroid invasion assay using a collagen matrix at 24 and 48 h. Centratherin treatment significantly impaired invasion: after 24 h at 0.5 μg/mL, the invaded area was reduced by ~ 50%, while at 1.5 and 2.5 μg/mL, it was reduced by ~ tenfold compared to controls (Fig. 2F, H). After 48 h, at 0.5 μg/mL, the reduction of the invaded area was ~ threefold reduction, whereas at higher concentrations it was reduced by ~ 24-fold (Fig. 2G, H). Collectively, these results demonstrate that centratherin disrupts GB cell morphology and effectively inhibits proliferation, motility, and invasiveness.

### Centratherin Disrupts Cytoskeletal Architecture and Promotes Formation of Intercellular Nanotubes in GB Cells

**Fig. 3:**
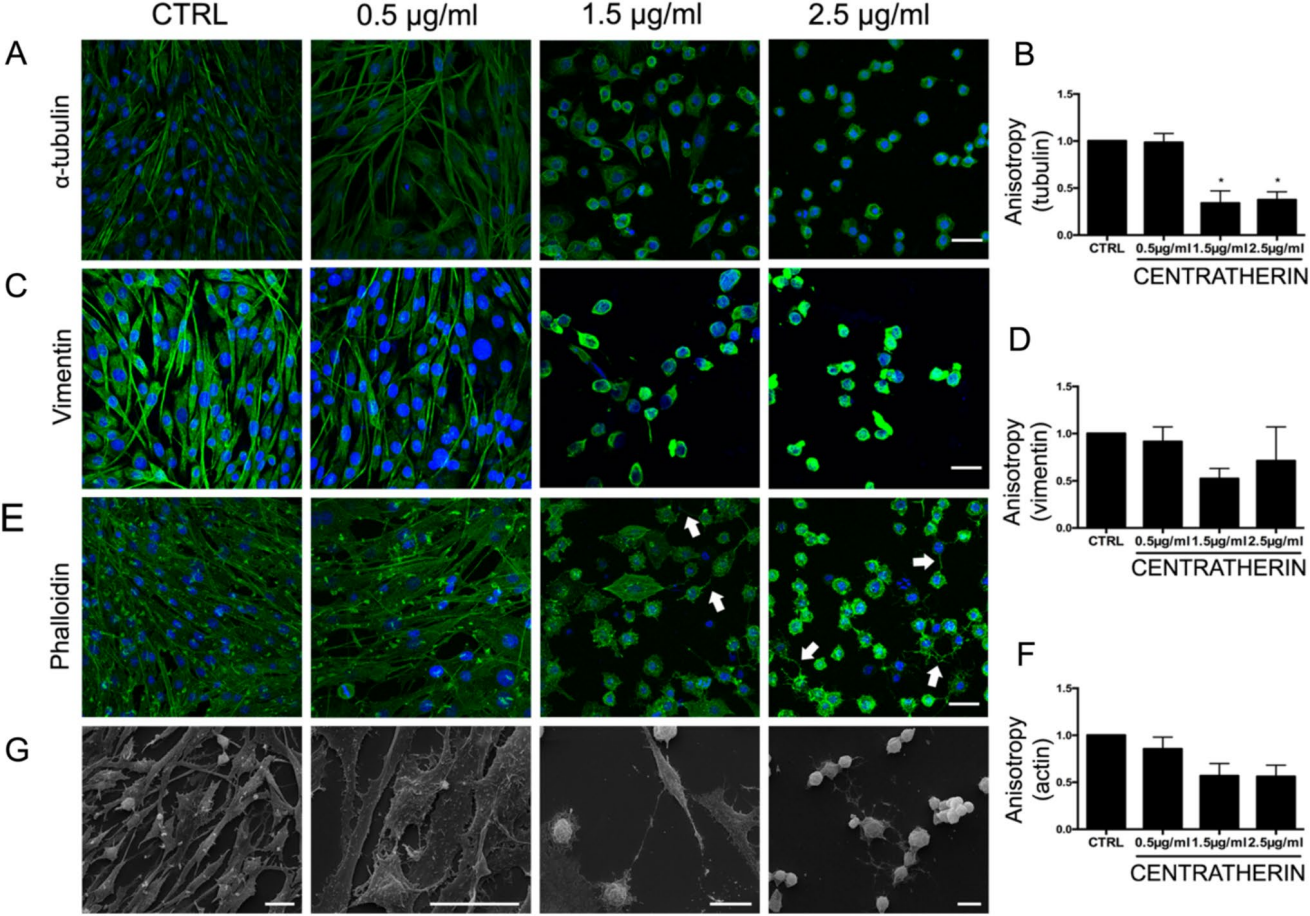
Centratherin disrupts cytoskeletal architecture and promotes the formation of intercellular nanotubes in glioblastoma cells. GBM02 cells treated with vehicle (CTRL) or centratherin (0.5, 1.5, or 2.5 µg/mL) for 24 h, stained for α-tubulin, vimentin, and actin (phalloidin), and analyzed for filament anisotropy. **A**, **C**, **E** Representative images show a progressive loss of filament organization with increasing centratherin concentration, **B**, **D**, **F** with quantification revealing a significant reduction in anisotropy of approximately 65% for tubulin and around 50–55% for vimentin and actin at 2.5 μg/mL compared to untreated controls. In addition, **E** phalloidin staining revealed actin-rich membrane protrusions emerging in cells treated with 1.5 and 2.5 μg/mL centratherin (white arrows), suggesting cytoskeletal remodeling. **G** Scanning electron microscopy confirmed the presence of elongated membrane structures consistent with intercellular nanotubes connecting adjacent cells. Together, these findings indicate that centratherin induces a profound disorganization of the cytoskeleton and nanotubes connecting adjacent cells. Values represent the mean ± SEM (n = 3). Scale bars: 20 µm. Statistical significance: *p < 0.01 compared to control

Given the observed effects of centratherin on GB cell motility, proliferation, and morphology—processes tightly regulated by the cytoskeleton—we investigated its impact on cytoskeletal organization [26–28]. Tubulin, vimentin, and actin filaments were stained and analyzed using anisotropy, a quantitative metric of filament alignment and organization [23]. Treatment with 2.5 μg/mL centratherin significantly reduced cytoskeletal anisotropy by 65% for tubulin and by ~ 50–55% for vimentin and actin (Fig. 3A–F), indicating a substantial loss of cytoskeletal organization. Phalloidin staining revealed actin-based membrane protrusions in cells treated with 1.5 and 2.5 μg/mL (white arrows, Fig. 3E). Scanning electron microscopy confirmed these structures as intercellular nanotubes connecting neighboring cells (Fig. 3G), indicating centratherin-induced cytoskeletal remodeling.

### Centratherin Disrupts Cell Cycle Progression Without Inducing Phase-Specific Arrest

**Fig. 4:**
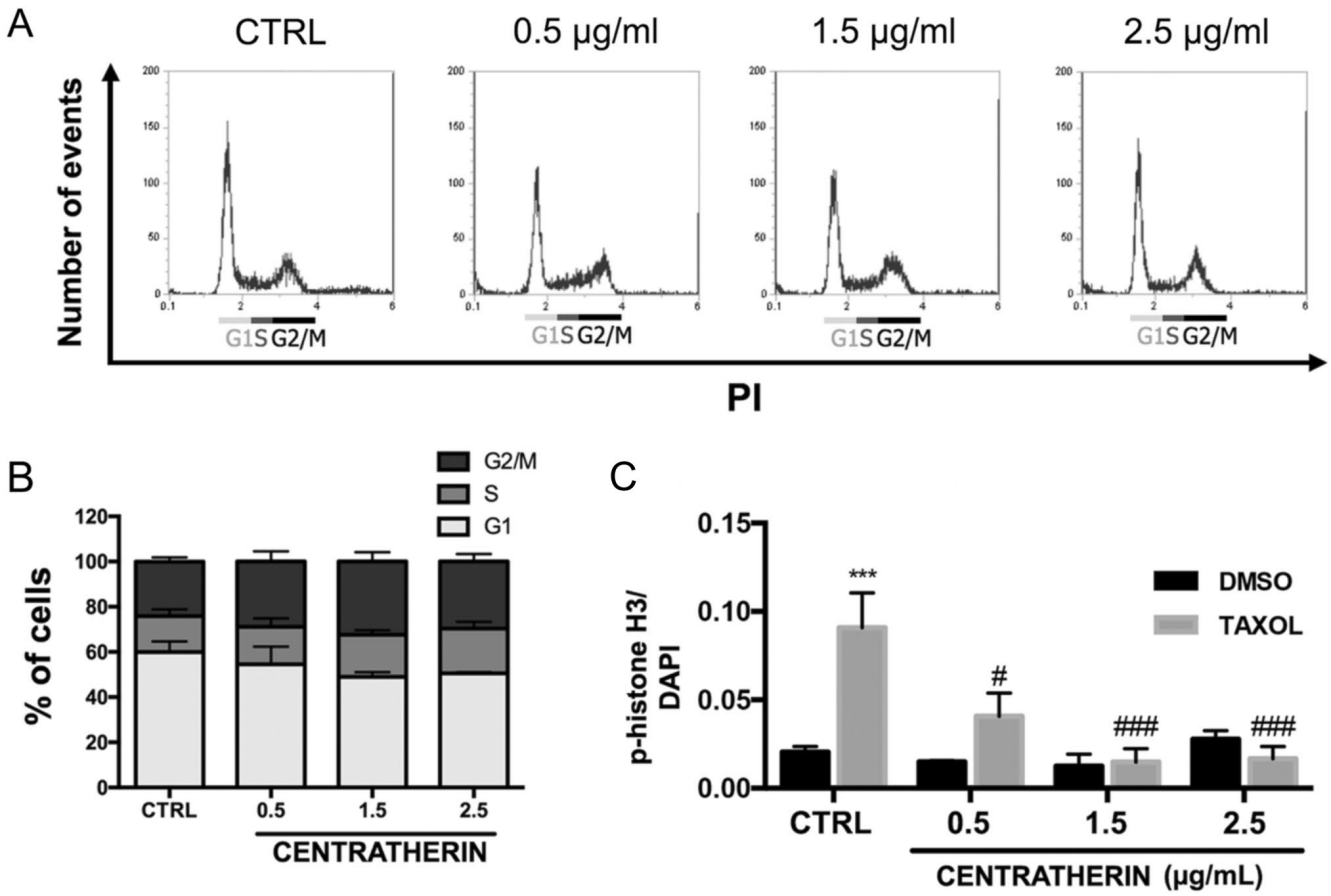
Centratherin interferes with cell cycle progression without phase-specific arrest. GBM02 cells treated with vehicle (CTRL) or centratherin (0.5, 1.5, or 2.5 µg/mL) for 24 h and analyzed by flow cytometry following propidium iodide (PI) staining. **A** Representative histograms and **B** quantification of cell distribution across G1, S, and G2/M phases revealed no significant changes among treatment groups, indicating that centratherin does not induce arrest at a specific cell cycle phase. To further explore this effect, **C** cells were pre-treated with centratherin for 24 h and then exposed to 1 μM taxol or vehicle (CTRL) for 3 h. Taxol treatment alone led to a significant accumulation of mitotic cells, as shown by phospho-histone H3 (pH3) staining. However, this taxol-induced mitotic accumulation was abolished in cells previously treated with centratherin, regardless of the centratherin concentration. These results suggest that centratherin interferes with cell cycle progression, likely by impairing the entry into or completion of mitosis, without producing classical phase-specific arrest. Values represent the mean ± SEM (n = 3). Statistical significance: ***p < 0.001 for comparisons between DMSO- and taxol-treated samples at the same centratherin concentration; #p < 0.05 and ###p < 0.001 for comparisons between taxol-treated cells and control

Time-lapse microscopy revealed that centratherin inhibits proliferation, as observed by the decrease in cytokinesis events (Fig. 2C, D and Videos S2–S4, compared to Video S1), prompting further investigation into its impact on cell cycle regulation. Flow cytometry after propidium iodide (PI) staining showed no significant changes in cell cycle phase distribution following treatment, indicating the absence of classical phase-specific arrest (Fig. 4A, B). Since videomicroscopy revealed a strong reduction in proliferation, but PI flow cytometry cell cycle analysis showed no accumulation in a specific phase, we next asked whether centratherin-treated cells progress through the cell cycle similarly to the control cells. To further explore its impact, GB cells were pretreated with centratherin for 24 h, followed by a 3 h exposure to taxol, a microtubule-stabilizing agent that arrests cells in mitosis [29]. GB cells were pre-treated with centratherin for 24 h, followed by a 3 h treatment with taxol. As expected, taxol increased the proportion of mitotic cells in the control group, as indicated by phospho-histone H3 staining. However, in centratherin-treated cells, this mitotic accumulation was absent, regardless of taxol exposure (Fig. 4C). These results suggest that centratherin interferes with normal cell cycle progression, potentially disrupting entry into or completion of mitosis without inducing phase-specific arrest.

### Centratherin Induces Necrotic Cell Death and DNA Damage in GB Cells

**Fig. 5:**
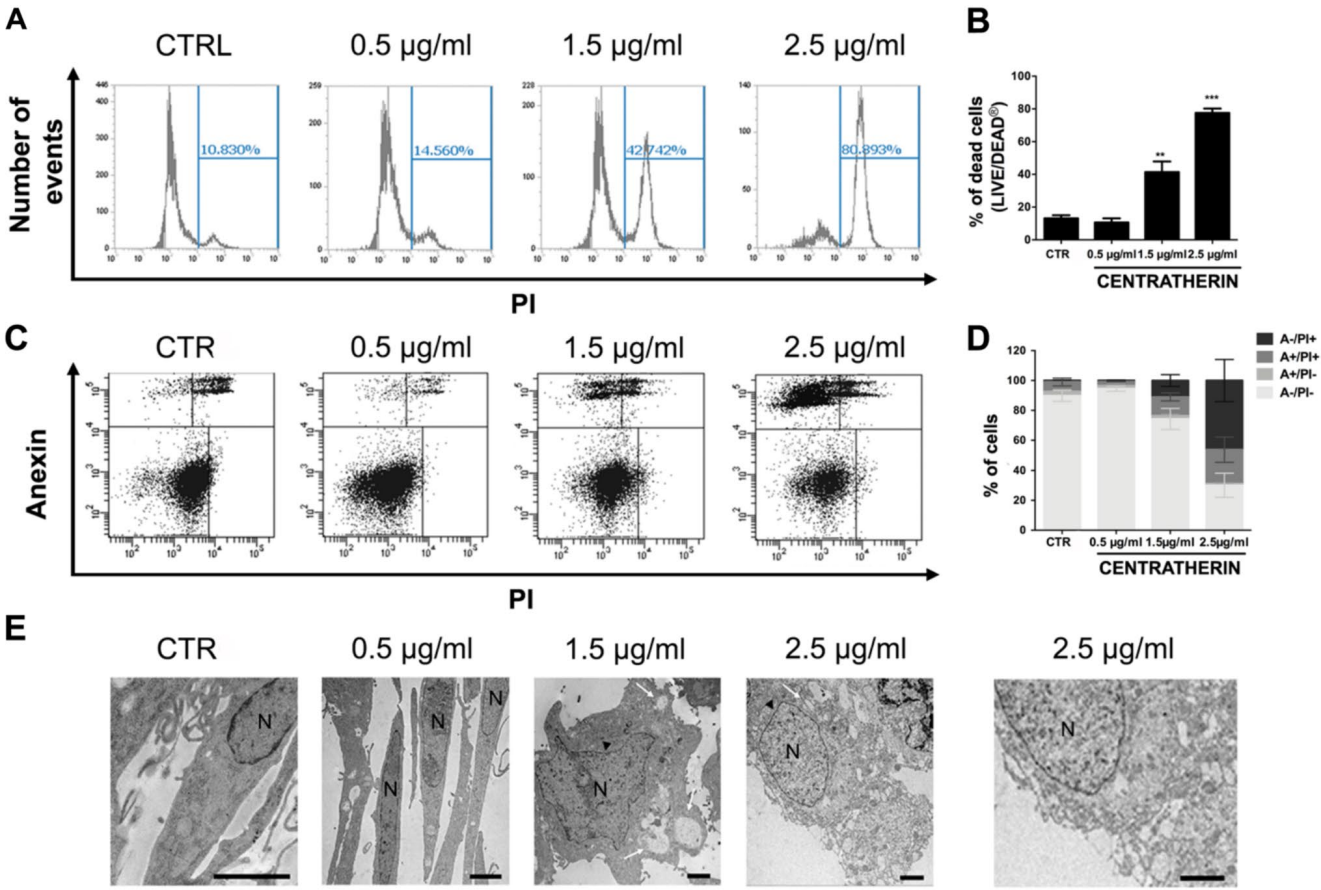
Centratherin induces necrotic cell death and DNA damage in glioblastoma cells. GBM02 cells treated with vehicle (CTR) or centratherin at 0.5, 1.5, or 2.5 μg/mL for 24 h to assess cell viability and the mechanism of cell death. **A**, **B** LIVE/DEAD staining shows increased cell death at 1.5 µg/mL (41%) and 2.5 µg/mL (77%). To determine the nature of this cell death, **C**, **D** Annexin V/PI staining was performed. At 1.5 μg/mL, 8.8% of cells were Annexin V/PI double-positive and 10.6% were PI-positive only; at 2.5 μg/mL, 17.4% of cells were double-positive and 41.6% were PI-positive alone, suggesting a mixed cell death phenotype with a predominance of necrotic features. **E** Transmission electron microscopy revealed cytoplasmic vacuolization, loss of plasma membrane integrity, and intact nuclei, with no signs of chromatin condensation or apoptotic bodies, confirming the necrotic morphology; N nuclei, arrowhead preserved nuclear morphology, white arrows cytoplasmic vacuolization. Values represent mean ± SEM (n = 3). Statistical significance: *p < 0.05, **p < 0.01 compared to control. Scale bars: 2 µm

**Fig. 6:**
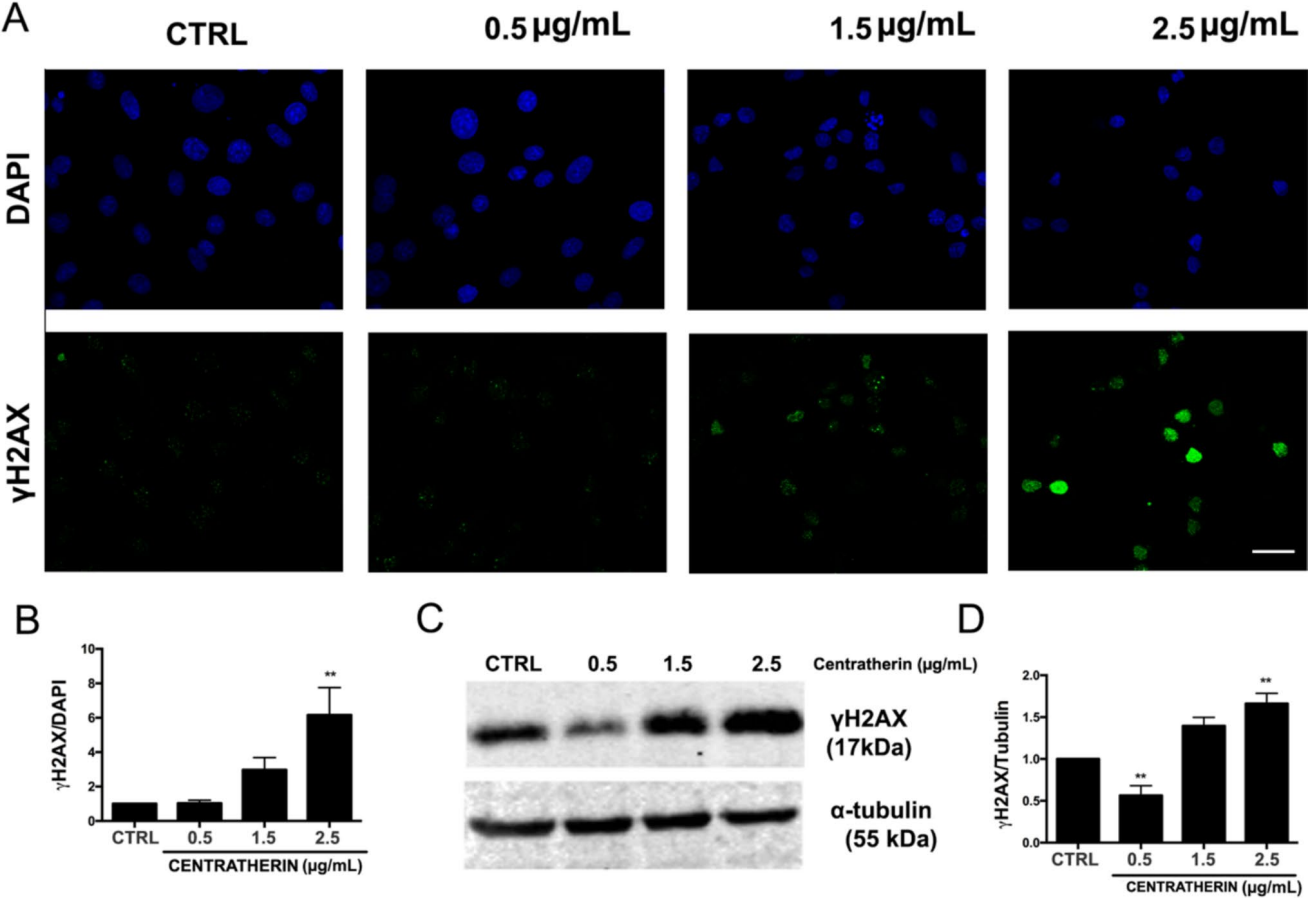
Centratherin induces DNA double-strand breaks in glioblastoma cells. GBM02 cells treated with vehicle (CTR) or centratherin (0.5, 1.5, or 2.5 μg/mL) for 24 h and analyzed for the presence of double-strand breaks using γH2AX staining. **A** γH2AX staining shows increased positive nuclei at 2.5 µg/mL compared to controls, **B** with quantification confirming a significant rise in the percentage of γH2AX-positive cells. **C** Western blot analysis corroborated these findings, showing increased γH2AX levels in cells treated with 2.5 μg/mL centratherin, and densitometric quantification demonstrated a robust elevation relative to the untreated group (**D**). These results indicate that centratherin treatment leads to DNA damage, contributing to the observed necrotic cell death phenotype. Values represent the mean ± SEM (n = 3). Statistical significance: **p < 0.01 compared to control. Scale bar: 25 µm

It has been proposed that intercellular nanotubes may facilitate chemoresistance by mediating drug efflux [30]. Given that centratherin induces nanotube formation in GB cells, we investigated whether it also promotes cell death. LIVE/DEAD assays revealed no significant cell death at 0.5 μg/mL, but treatment with 1.5 and 2.5 μg/mL increased death rates to 41 and 77%, respectively (Fig. 5A, B). Annexin V/PI staining indicated that at 1.5 μg/mL, 8.8% of cells were Annexin V/PI double-positive and 10.6% were PI-positive only, whereas at 2.5 μg/mL, 17.4% were double-positive and 41.6% were PI-positive only (Fig. 5C, D), consistent with necrotic or late apoptotic death. Transmission electron microscopy revealed hallmark features of necrosis—cytoplasmic vacuolization, membrane rupture, and preserved nuclear morphology—without classical apoptotic morphology [31] (Fig. 5E). Necrostatin-1 failed to prevent death, indicating a RIP1-independent necrotic pathway (Fig. S2A–C). Since necrosis can result from DNA damage [32], we assessed the presence of DNA double-strand breaks. Immunofluorescence and western blot analysis showed increased γH2AX levels following 2.5 μg/mL treatment, confirming induction of DNA double-strand breaks (Fig. 6A–D).

### Centratherin is Cytotoxic to GB Stem-Like Cells and Compatible with Temozolomide

**Fig. 7:**
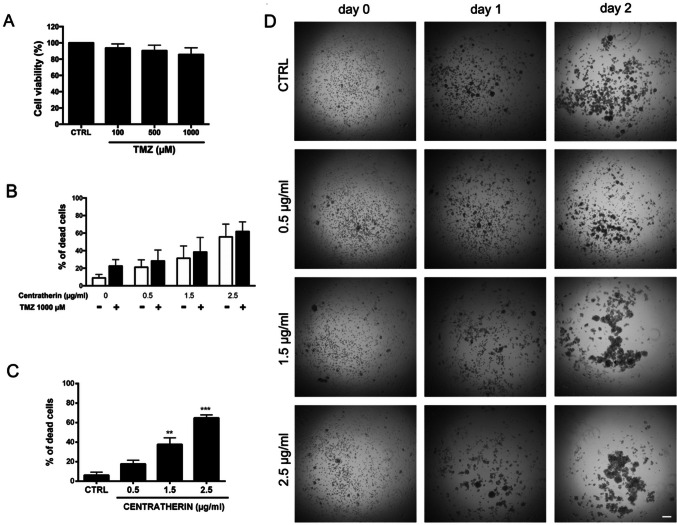
Centratherin is cytotoxic to GB stem-like cells and compatible with temozolomide. **A** Cell viability (MTT assay) after treatment with TMZ at 100, 500, and 1000 μM for 24 h in GBM02 cells and normalized to the vehicle control. Data represent the mean ± SEM of three independent experiments performed in triplicate. **B** Flow cytometry analysis using the LIVE/DEAD® dye of GBM02 cells treated for 24 h with centraterin (0.5, 1.5, and 2.5 μg/mL) and 1000 μM TMZ or vehicle. Graph shows the percentage of dead cells for each experimental condition. Data represent the mean ± SEM of three independent experiments. **C** and **D** Glioblastoma stem-like cells (GSCs) treated with vehicle (CTRL) or centratherin (0.5, 1.5, or 2.5 µg/mL) for 24 h. **C** % Dead cells evaluated by trypan blue cell count. **D** Representative images of centratherin treated GSCs. Scale bar: 200 µm

Considering the potential use of centratherin alongside standard GB therapy, we tested its compatibility with temozolomide (TMZ) [33, 34]. To assess whether centratherin can be used alongside TMZ, we first evaluated the effect of TMZ alone on GBM02 cells using the MTT assay. TMZ alone (100–1000 μM, 24 h) did not significantly reduce GBM02 viability (Fig. 7A). Combined treatment with centratherin (0.5–2.5 μg/mL) and 1000 μM TMZ produced cytotoxic effects similar to centratherin alone, indicating neither synergy nor antagonism (Fig. 7B). These findings suggest that centratherin can be co-administered with TMZ without loss of efficacy. Given that glioblastoma stem-like cells (GSCs) contribute to recurrence and TMZ resistance [35], we investigated centratherin’s effect on this population. Our group has previously demonstrated that GB cells can acquire or lose stem-like properties in response to environmental cues simply by changing the culture conditions [36]. Based on this phenomenon, GBM02 cells were cultured in a serum-free defined medium to induce the acquisition of stem-like features, as described by Balça-Silva et al. [36]. After 2 weeks under these conditions, GBM02-derived GSCs were treated with different concentrations of centratherin. Treatment with 1.5 and 2.5 μg/mL centratherin induced 37.7% and 64.8% cell death, respectively (Fig. 7C), demonstrating its cytotoxicity against GSCs.

## Discussion

GB is a highly aggressive and heterogeneous primary tumor of the central nervous system, characterized by rapid proliferation, high migratory and invasive capacity, and marked resistance to cell death. These features substantially limit the efficacy of current therapeutic approaches and contribute to the poor prognosis, with a median survival of approximately 14–15 months after diagnosis despite maximal surgical resection, radiotherapy, and chemotherapy with temozolomide (TMZ) [2, 3]. In this context, there is increasing interest in natural products—particularly sesquiterpene lactones—as alternative or complementary strategies for GB therapy, owing to their pleiotropic biological effects and selective cytotoxicity toward tumor cells [37 – 39].

In this study, we investigated the antitumor properties of centratherin, a sesquiterpene lactone isolated from Eremanthus crotonoides, in established and patient-derived GB models. Our results demonstrate that centratherin exerts a multifaceted inhibitory effect on GB cell viability, proliferation, motility, invasion, and cytoskeletal architecture, and induced cell death, predominantly through necrosis. Importantly, these effects were predominantly observed in tumor cells, while healthy human astrocytes showed limited sensitivity to centratherin under identical conditions. Although our evaluation of astrocytes was restricted to viability assays, the apparent selectivity of centratherin remains encouraging feature, in line with reports for other sesquiterpene lactones such as alantolactone, iso-seco-tanapartolide, and brevilin A [7, 40, 41].

Centratherin reduced the viability of both established (U87) and patient-derived (GBM02, GBM95) GB cell lines in a concentration-dependent manner, with IC₅₀ values consistent with prior reports [11]. Sensitivity differences between cell lines may reflect genetic heterogeneity. For instance, the differential sensitivity between U87 (wild-type *TP53*) and U251 (mutant *TP53*) cells has been previously associated with differential responses to chemotherapeutic agents such as etoposide [42, 43], suggesting that *TP53* status may also influence centratherin efficacy.

To investigate whether centratherin’s reduction in cell viability was due solely to cytotoxicity or also involved modulation of specific tumorigenic properties, we analyzed its effects on GB cell behavior at sub-lethal concentrations. Videomicroscopy analysis revealed that centratherin profoundly altered cell morphology, reducing proliferation and motility even at 0.5 μg/mL—concentrations that did not significantly induce cell death. These findings suggest that centratherin may interfere with key regulatory pathways governing cytoskeletal dynamics and cell cycle progression. Indeed, centratherin disrupted actin, vimentin, and tubulin organization, reduced filament anisotropy, and induced actin-rich protrusions and intercellular nanotubes. This disruption likely underlies the observed impairments in cell division, morphology, and motility. Additionally, we observed the emergence of actin-rich protrusions and intercellular nanotubes—structures implicated in intercellular communication and, in some contexts, chemoresistance [30, 44, 45]. Whether nanotube formation in this context represents a compensatory survival mechanism or a consequence of cytoskeletal destabilization warrants further investigation.

Regarding proliferation, centratherin did not induce classical cell cycle arrest, as assessed by propidium iodide-based flow cytometry. However, centratherin-treated cells exhibited reduced accumulation in mitosis following taxol exposure, suggesting a generalized slowing of cell cycle progression rather than arrest at a specific phase. This phenotype is reminiscent of other regulatory mechanisms that reduce proliferation by impairing cell cycle kinetics without altering phase distribution, such as PTEN overexpression or CD32-mediated modulation [46, 47].

Consistent with its effects on morphology and cytoskeleton, centratherin also reduced the invasive capacity of GB cells in a collagen-based 3D spheroid assay. The pronounced inhibition of invasion, particularly at 1.5 and 2.5 μg/mL, suggests that centratherin may suppress both cytoskeletal dynamics and extracellular matrix interaction—key processes for GB infiltration into surrounding brain tissue. While the assay does not distinguish between anti-proliferative and anti-invasive effects, the inhibition observed at non-cytotoxic doses supports a direct role in invasion suppression.

Importantly, centratherin induced cell death predominantly by necrosis rather than apoptosis. Flow cytometry analysis with Annexin V/PI, along with ultrastructural analysis by transmission electron microscopy, revealed features consistent with necrosis, such as vacuolization and membrane rupture without chromatin condensation. Additionally, centratherin-induced death was not attenuated by the RIP1 inhibitor necrostatin-1, and RIP1 expression levels were unchanged, excluding necroptosis as the underlying mechanism. This is particularly relevant for GB, which is often resistant to apoptosis due to elevated anti-apoptotic proteins such as Bcl-2, Bcl-XL, and IAPs [48, 49). Notably, centratherin treatment increased γH2AX levels, indicating DNA double-strand breaks, a potential trigger for regulated necrosis pathways. Similar DNA damage-associated necrosis has been reported for other sesquiterpene lactones, including artesunate and dehydroleucodine [50, 51]. While RIP1-mediated necroptosis was excluded as seen in Fig. S2, other necrotic subroutines, such as parthanatos or mitochondrial permeability transition pore-driven necrosis, remain possible [52–54].

No synergistic interaction was observed between centratherin and TMZ under the tested conditions, despite reports that other natural compounds (e.g., resveratrol, quercetin, parthenolide) can sensitize GB cells to TMZ [55–57]. The absence of antagonism, however, supports the potential for co-administration, particularly given centratherin’s distinct mechanism of action.

Centratherin’s ability to induce necrosis, damage DNA, and impair cell motility, all while apparently sparing healthy astrocytes, makes it a promising compound for further investigation. Importantly, centratherin was also cytotoxic to GSCs, a therapy-resistant subpopulation implicated in tumor recurrence [34]. This activity, combined with its selective toxicity and multifaceted inhibition of malignant phenotypes, underscores centratherin’s potential as a therapeutic candidate in recurrent GB settings.

In conclusion, centratherin exhibits potent and selective antitumor activity against GB cells, impairing viability, proliferation, motility, and invasion; disrupting cytoskeletal integrity; inducing DNA damage; and promoting necrotic cell death. These findings provide strong preclinical rationale for further investigation of centratherin, including elucidation of its molecular targets, evaluation of its in vivo efficacy and safety, and exploration of its potential within combinatorial treatment regimens.

## Supplementary Information

Below is the link to the electronic supplementary material.

Supplementary file1 (AVI 3236 KB)

Supplementary file2 (AVI 2190 KB)

Supplementary file3 (AVI 2918 KB)

Supplementary file4 (AVI 3175 KB)

Supplementary file5 (DOCX 209 KB)

## Data Availability

No datasets were generated or analysed during the current study.
