# Supplementary material for: Centratherin Exhibits Antitumor Activity Against Glioblastoma Cells
Source: Neurochem Res. 2026 Feb 7;51(1):67. doi: 10.1007/s11064-025-04659-6 (PMC12882865; doi:10.1007/s11064-025-04659-6)
Supplement: Supplementary file 5 — Supplementary file5 (DOCX 209 KB) [file 11064_2025_4659_MOESM5_ESM.docx]

Supplementary Material:


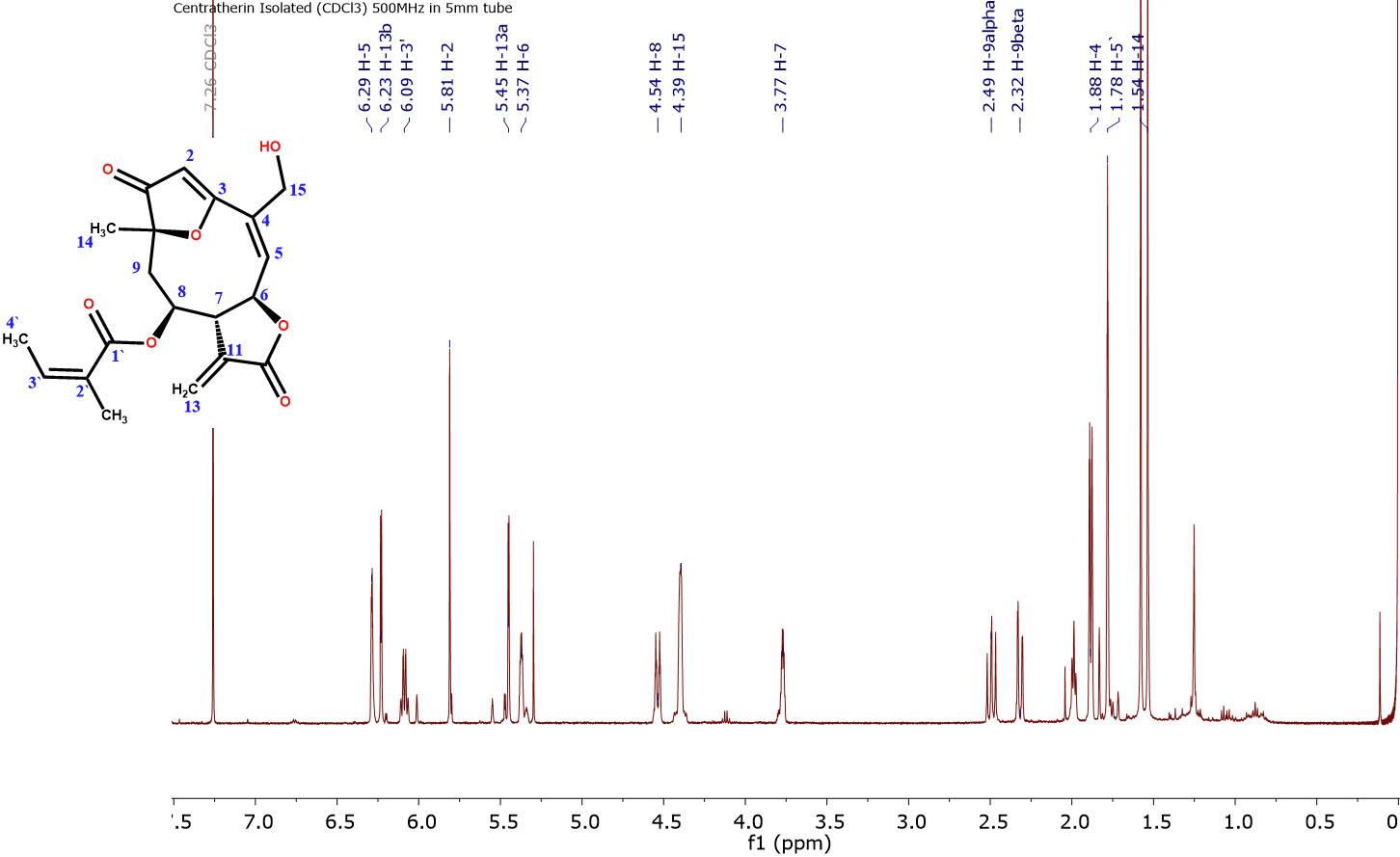


**Figure S1.** **^1^H NMR spectrum of the purified centratherin** (CDCl₃, 499.77 MHz at 25 °C). The characteristic resonances are consistent with literature data and confirm the expected structure. The absence of extraneous signals indicates a purity greater than 95%. Experimental parameters: 1D experiment (pulse sequence = s2pul), 8 scans, relaxation delay = 1.0 s, pulse width = 3.75 µs. Instrument: Varian VNMR (p5mmautoX probe).


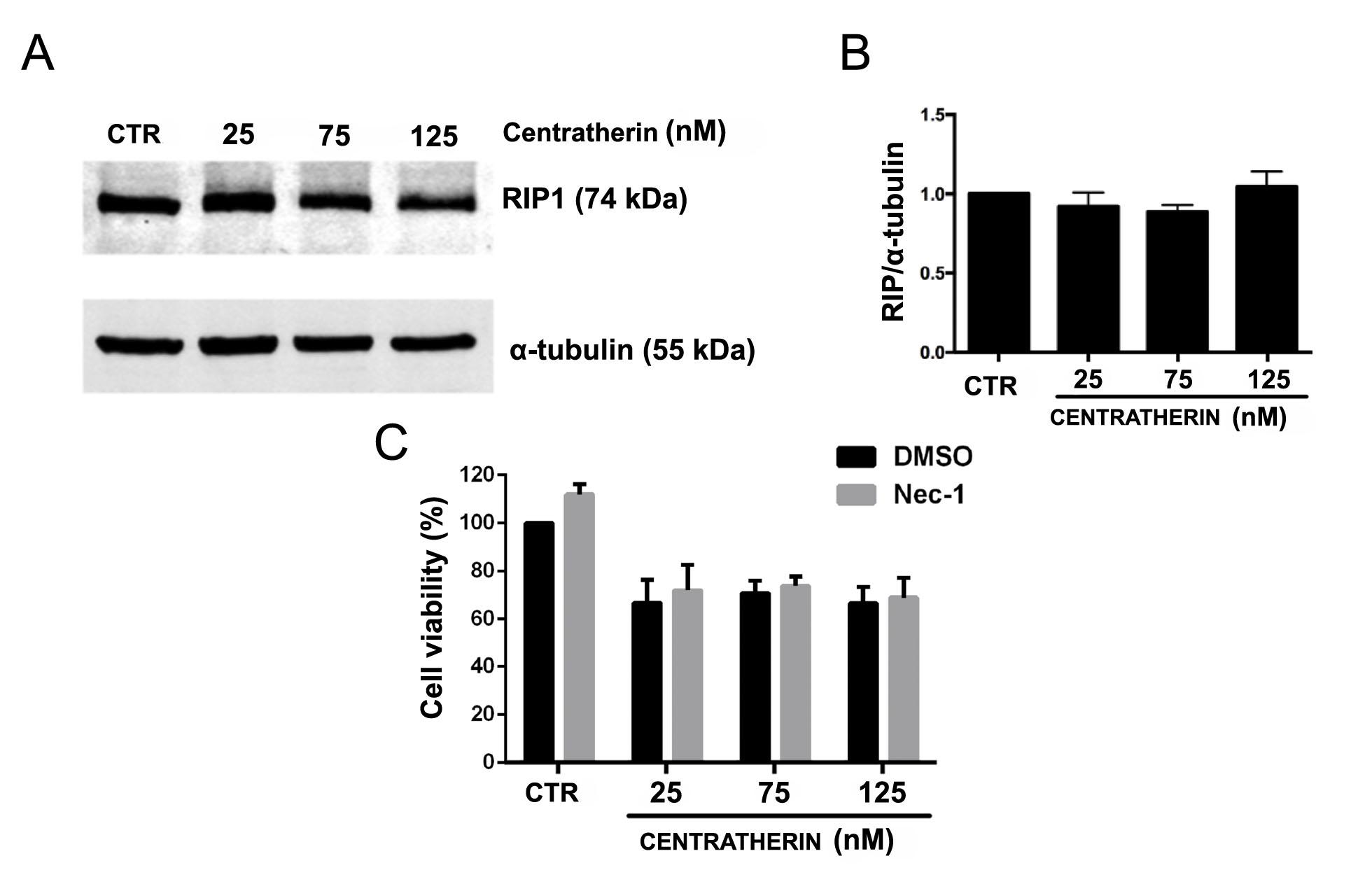


**Figure S2. Centratherin triggers RIP1-independent cell death.** GBM02 cells treated with vehicle (CTR) or centratherin (0.5, 1.5, or 2.5 μg/mL). (A) Representative Western blot of RIP1 and α-tubulin after 3 h. (B) RIP1 levels normalized to α-tubulin; mean ± SEM, n = 3. (C) Cell viability (MTT assay) after Nec-1 (100 μM, 1 h) or vehicle pretreatment, followed by centraterin for 24 h; mean ± SEM, n = 3, performed in triplicate.

**Video S1. Time-lapse microscopy recording GMB02 cells under the CTRL condition.**

**Video S2. Time-lapse microscopy of GBM02 cells following treatment with 0.5 μg/mL centratherin.**

**Video S3. Time-lapse microscopy of GBM02 cells following treatment with 1.5 μg/mL centratherin.**

**Video S4.** **Time-lapse microscopy of GBM02 cells following treatment with 2.5 μg/mL centratherin.**
